# The effect of antithrombin added to recombinant human-soluble thrombomodulin for severe community-acquired pneumonia-associated disseminated intravascular coagulation: a retrospective cohort study using a nationwide inpatient database

**DOI:** 10.1186/s40560-019-0419-8

**Published:** 2020-01-13

**Authors:** Jun Suzuki, Yusuke Sasabuchi, Shuji Hatakeyama, Hiroki Matsui, Teppei Sasahara, Yuji Morisawa, Toshiyuki Yamada, Hideo Yasunaga

**Affiliations:** 1 Division of Infectious Diseases, Jichi Medical University Hospital, 3311-1 Yakushiji, Shimotsuke, Tochigi 329-0498 Japan; 2 Data Science Center, Jichi Medical University, 3311-1 Yakushiji, Shimotsuke, Tochigi 329-0498 Japan; 3 Division of General Medicine, Jichi Medical University Hospital, 3311-1 Yakushiji, Shimotsuke, Tochigi 329-0498 Japan; 4 Department of Clinical Epidemiology and Health Economics, School of Public Health, The University of Tokyo, 7-3-1 Hongo, Bunkyo-ku, Tokyo 113-0033 Japan; 5 Department of Infection and Immunity, School of Medicine, Jichi Medical University, 3311-1 Yakushiji, Shimotsuke, Tochigi 329-0498 Japan; 6 Department of Clinical Laboratory Medicine, Jichi Medical University, 3311-1 Yakushiji, Shimotsuke, Tochigi 329-0498 Japan

**Keywords:** Pneumonia, Disseminated intravascular coagulation, Sepsis, Mortality

## Abstract

**Background:**

Studies showed potential benefits of recombinant human-soluble thrombomodulin (rhTM) and antithrombin for treating sepsis associated disseminated intravascular coagulation. However, benefits of their combination have been inconclusive.

**Methods:**

Using a nationwide inpatient database in Japan, we performed propensity-score matched analyses to compare outcomes between rhTM combined with antithrombin and rhTM alone for severe community-acquired pneumonia associated disseminated intravascular coagulation from July 2010 to March 2015. The outcomes included in-hospital mortality and requirement of red cell transfusion.

**Results:**

Propensity score matching created 189 pairs of patients who received rhTM combined with antithrombin or rhTM alone within 2 days of admission. There was no significant difference between the two groups for in-hospital mortality (40.2% vs. 45.5%). Patients treated with rhTM and antithrombin were more likely to require red cell transfusion than those treated with rhTM alone (37.0% vs. 25.9%).

**Conclusions:**

Compared with rhTM alone, combination of rhTM with antithrombin for severe community-acquired pneumonia-associated disseminated intravascular coagulation may be ineffective for reducing mortality and may increase bleeding.

## Background

Disseminated intravascular coagulation (DIC) is one of the major complications associated with sepsis [1], and sepsis patients with DIC have higher mortality than those without DIC [2]. The reported mortality of severe pneumonia with DIC ranges from 35 to 46% [3, 4]. Strategies for treating sepsis-associated DIC differ among the recent guidelines in several countries [5–8].

Recombinant human-soluble thrombomodulin (rhTM) plays an important role in inactivating coagulation [9]. In Japan, approximately 40 to 60% in patients with sepsis-induced DIC received rhTM [10, 11]. A previous Japanese randomized controlled trial (RCT) demonstrated that rhTM improved recovery from DIC [12], and a systematic review and meta-analysis showed that rhTM was associated with lower mortality in patients with sepsis-associated DIC [13, 14]. In response to these results, the Japanese guidelines recommend rhTM for sepsis-associated DIC [5]. However, the latest RCT and Japanese Diagnosis Procedure Combination database study showed that rhTM did not reduce morality in patients with sepsis-induced DIC [4, 15]. These results suggest that rhTM monotherapy may not be efficient for sepsis-induced DIC.

Antithrombin (AT) is also used as an anticoagulant in patients with sepsis-associated DIC, and rhTM is often used with AT for treatment of sepsis-associated DIC in Japan [16–19], and AT added to rhTM may reduce mortality for patients with sepsis induced DIC [20]. One previous study showed that AT added to rhTM was associated with lower mortality [19]. On the other hand, two previous studies showed no significant difference in mortality between the combination therapy and rhTM monotherapy in patients with sepsis-associated DIC [16, 18]. Therefore, the effect of the combination of rhTM and AT therapy is still controversial. In addition, combination of rhTM and AT may potentially cause harm in terms of increased bleeding, because rhTM and AT were each reported to be independently associated with bleeding complications [12, 21, 22].

Therefore, the purpose of this study was to compare in-hospital mortality and necessity for red cell transfusion between rhTM combined with AT and rhTM alone in patients with severe community-acquired pneumonia-associated DIC, using a Japanese national inpatient database.

## Methods

### Data source

Data for this study were abstracted from the Japanese Diagnosis Procedure Combination database. The database includes data for approximately 7 million inpatients per year from more than 1000 acute-care hospitals in Japan and represents approximately 50% of all discharges from acute-care hospitals in Japan.

The database includes the following data: hospital identification number, age, sex, primary diagnosis, comorbidities at admission, post-admission complications during hospitalization, dates of hospital admission and discharge, and discharge status (dead or alive). The primary diagnosis, comorbidities at admission, and post-admission complications during hospitalization are recorded with International Classification of Diseases 10th Revision (ICD-10) codes and text written in Japanese. The database also includes dates of surgeries and drug prescriptions [23–25].

### Patient data

We identified patients with severe community-acquired pneumonia associated with DIC between July 2010 and March 2015. We included patients diagnosed with sepsis, severe pneumonia, and DIC. Sepsis was defined as any bacterial or fungal infection at admission based on the Angus criteria [26] (Additional file 1: Table S1). A previous study validated the definition of sepsis by the Angus criteria, using the same database [27]. Pneumonia was identified by the ICD-10 codes listed in Additional file 1: Table S2. Severe pneumonia was defined as patients who required vasopressors and/or mechanical ventilation within 2 days after admission, according to the Infectious Diseases Society of America/American Thoracic Society Consensus guidelines [28]. DIC at admission was identified by ICD-10 code D65. A previous study validated the definition of DIC based on the criteria of Japanese Association for Acute Medicine, using the same database [27].

The exclusion criteria were as follows [3, 4, 29]: (1) age < 18 years, (2) pregnancy, (3) no administration of antibiotics within 2 days of admission, (4) non-sepsis-associated DIC such as trauma, vasculitis, aortic aneurysm, malignancy, obstetric complication, burn, severe toxic or immunological reaction, pancreatitis, heat stroke, rhabdomyolysis, malignant syndrome, and fat embolism, (5) intracerebral arteriovenous malformation, (6) congenital AT deficiency, (7) human immunodeficiency virus infection or acquired immunodeficiency syndrome, (8) bleeding of esophageal varices, (9) hepatic failure, (10) red cell transfusion within 2 days of admission, and (11) discharge within 2 days of admission.

### Study variables

The exposure of interest was combination therapy with rhTM and AT within 2 days of admission. The reference group was defined as patients who received rhTM alone within 2 days of admission.

Other variables included age, sex, intensive care unit (ICU) or high care unit (HCU) admission within 2 days of admission, hospital type (academic or nonacademic), hospital volume of patients with severe community-acquired pneumonia, and Japan coma scale (JCS). Hospital volume of patients with severe community-acquired pneumonia was defined as the annual average number of patients with severe community-acquired pneumonia in each hospital, according to the Infectious Diseases Society of America/American Thoracic Society Consensus guidelines [28]. JCS scores were categorized into four groups [30, 31]: JCS 0, patients with alert consciousness; JCS 1–3, patients with delirium; JCS 10–30, patients with somnolence; and JCS 100–300, patients with coma. JCS scores are well correlated with Glasgow Coma Scale scores [32]. We also evaluated the following procedures within 2 days of admission: use of mechanical ventilation, intermittent and continuous renal replacement therapy, polymyxin B hemoperfusion, bacterial culture collection, bronchoscopy, vasopressors including noradrenaline and dopamine, hydrocortisone, intravenous immunoglobulin, heparin, low-molecular-weight heparin, danaparoid, sivelestat sodium, platelet concentrates, fresh-frozen plasma transfusion, and initial use of antibiotics (ampicillin, ampicillin/sulbactam, piperacillin/tazobactam, first-generation cephalosporin, third-generation cephalosporin without effect for *Pseudomonas aeruginosa*, fourth-generation cephalosporin, carbapenem, aminoglycoside, fluoroquinolone, macrolide, tetracycline, clindamycin, anti-methicillin-resistant *Staphylococcus aureus* drugs, antifungal drugs). Comorbidity of myocardial infarction, penicillin, second-generation cephalosporin, or third-generation cephalosporin with effect for *P. aeruginosa* were not analyzed because few patients had the comorbidity or these antibiotics.

### Outcomes

The outcomes were in-hospital mortality and need for red cell transfusion during hospitalization.

### Statistical analysis

Descriptive statistics were presented before and after propensity score matching. Continuous variables were presented as mean with standard deviation. Categorical variables were presented as numbers with percentages.

One-to-one propensity score matching was used to adjust for differences in baseline characteristics and severity of conditions on admission between the combination therapy group and the monotherapy group. The probability that a patient received combination therapy was modeled for confounders in the following characteristics: age, sex, ICU admission, HCU admission, hospital type (academic), hospital volume, consciousness level. comorbidities at admission, use of mechanical ventilation, intermittent and continuous renal replacement therapy, polymyxin B hemoperfusion, bacterial culture collection, bronchoscopy, vasopressors including noradrenaline and dopamine, hydrocortisone, intravenous immunoglobulin, heparin, low-molecular-weight heparin, danaparoid, sivelestat sodium, platelet concentrates, fresh-frozen plasma transfusion, and initial use of antibiotics. Differences between the combination therapy group and the monotherapy group before and after propensity score matching were assessed by standardized mean differences [33]. Absolute standardized mean differences of less than 0.1 were considered negligible imbalances in baseline characteristics between the two groups [34]. Fisher’s exact test was used to compare in-hospital mortality and proportion of patients who required red cell transfusion between the two groups. A *p* value of less than 0.05 was considered statistically significant. Propensity score matching was performed using the “matching” package in statistical software R version 3.1.3 (The R Foundation, Vienna, Austria). All other analyses were performed using IBM SPSS version 22 (IBM SPSS, Armonk, NY).

## Results

### Study population

**Fig. 1:**
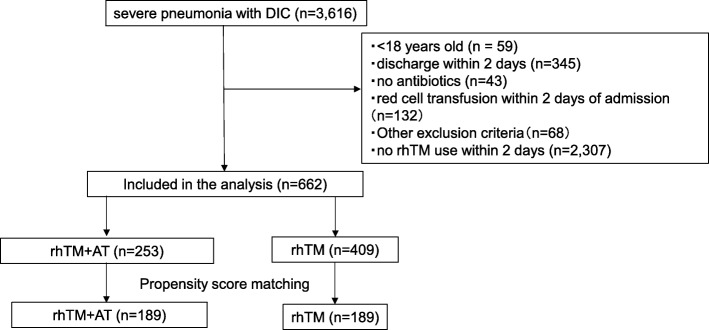
Patient selection chart. Abbreviations: DIC, disseminated intravascular coagulation; rhTM, recombinant human-soluble thrombomodulin; AT, antithrombin

We excluded approximately 60% (2307/3616) patients with severe community-acquired pneumonia-associated DIC who did not use rhTM. After the application of the inclusion and exclusion criteria, we identified 662 eligible patients during the study period. The combination therapy group included 253 patients and the monotherapy group included 409 patients. After one-to-one propensity score matching, 189 pairs were created (Fig. 1).

Table 1 shows the baseline characteristics in the unmatched and propensity score-matched groups. After propensity score matching, the patient distributions were well-balanced between the two groups.

**Table 1:** Baseline patient characteristics in the unmatched and propensity-matched groups

	Unmatched group			Propensity-matched group		
	rhTM+AT	rhTM	SMD	rhTM+AT	rhTM	SMD
	*n* = 253	*n* = 409		*n* = 189	*n* = 189	
Age, mean (SD)	71.7 (13.6)	73.6 (13.5)	0.134	71.2 (14.0)	72.0 (14.2)	0.057
Sex (male), *n* (%)	86 (34.0)	125 (30.6)	0.073	59 (31.2)	60 (31.7)	0.011
Hospital type (academic), *n* (%)	100 (39.5)	89 (21.8)	0.393	65 (34.4)	62 (32.8)	0.034
Hospital volume (cases/year), mean (SD)	120.1 (46.5)	106.4 (57.3)	0.263	113.1 (42.7)	114.9 (57.5)	0.035
ICU admission, *n* (%)	76 (30.0)	114 (27.9)	0.048	64 (33.9)	58 (30.7)	0.068
HCU admission, *n* (%)	19 (7.5)	24 (5.9)	0.066	15 (7.9)	15 (7.9)	< 0.001
Comorbidity, *n* (%)						
Congestive heart failure	26 (10.3)	57 (13.9)	0.112	21 (11.1)	20 (10.6)	0.017
Peripheral vascular disease	3 (1.2)	7 (1.7)	0.044	3 (1.6)	3 (1.6)	< 0.001
Cerebrovascular disease	12 (4.7)	20 (4.9)	0.007	9 (4.8)	7 (3.7)	0.053
Dementia	6 (2.4)	9 (2.2)	0.011	4 (2.1)	6 (2.1)	< 0.001
Chronic pulmonary disease	13 (5.1)	26 (6.4)	0.052	11 (5.8)	12 (6.3)	0.022
Rheumatologic disease	8 (3.2)	10 (2.4)	0.043	4 (2.1)	5 (2.6)	0.035
Peptic ulcer	6 (2.4)	10 (2.4)	0.005	6 (3.2)	5 (2.6)	0.031
Mild liver disease	9 (3.6)	15 (3.7)	0.006	6 (3.2)	7 (3.7)	0.029
Diabetes without chronic complications	27 (10.7)	43 (10.5)	0.005	21 (11.1)	20 (10.6)	0.017
Diabetes with chronic complications	8 (3.2)	14 (3.4)	0.015	8 (4.2)	8 (4.2)	< 0.001
Renal disease	20 (7.9)	28 (6.8)	0.041	17 (9.0)	15 (7.9)	0.038
Consciousness level, *n* (%)						
Alert	80 (31.6)	192 (46.9)	0.318	73 (38.6)	68 (36.0)	0.055
Delirium	71 (28.1)	83 (20.3)	0.182	49 (25.9)	53 (28.0)	0.048
Somnolence	47 (18.6)	47 (11.5)	0.199	26 (13.8)	24 (12.7)	0.031
Coma	52 (20.6)	70 (17.1)	0.088	38 (20.1)	40 (21.2)	0.026
Intervention, *n* (%)						
Mechanical ventilation	203 (80.2)	268 (65.5)	0.336	144 (76.2)	141 (74.6)	0.037
Intermittent renal replacement therapy	62 (24.5)	53 (13.0)	0.299	37 (19.6)	41 (21.7)	0.052
Continuous renal replacement therapy	10 (4.0)	11 (2.7)	0.071	7 (3.7)	6 (3.2)	0.029
Polymyxin B hemoperfusion	43 (17.0)	39 (9.5)	0.221	24 (12.7)	27 (14.3)	0.046
Bacterial culture collection	244 (96.4)	382 (93.4)	0.139	180 (95.2)	179 (94.7)	0.024
Bronchoscopy	20 (7.9)	16 (3.9)	0.17	12 (6.3)	12 (6.3)	< 0.001
Catecholamine, *n* (%)						
Noradrenaline	199 (78.7)	232 (56.7)	0.482	139 (73.5)	138 (73.0)	0.012
Dopamine	99 (39.1)	213 (52.1)	0.262	80 (42.3)	84 (44.4)	0.043
Platelet transfusion, *n* (%)	30 (11.9)	32 (7.8)	0.136	20 (10.6)	18 (9.5)	0.035
Fresh-frozen plasma transfusion, *n* (%)	28 (11.1)	15 (3.7)	0.286	10 (5.3)	14 (7.4)	0.087
Immunoglobulin, *n* (%)	136 (53.8)	125 (30.6)	0.483	91 (48.1)	85 (45.0)	0.064
Heparin, *n* (%)	209 (82.6)	242 (59.2)	0.534	148 (78.3)	152 (80.4)	0.052
Danaparoid, *n* (%)	5 (2.0)	8 (2.0)	0.001	5 (2.6)	4 (2.1)	0.035
Sivelestat sodium, *n* (%)	81 (32.0)	101 (24.7)	0.163	61 (32.3)	62 (32.8)	0.011
Low-molecular-weight heparin, *n* (%)	7 (2.8)	7 (1.7)	0.071	6 (3.2)	5 (2.6)	0.031
Hydrocortisone, *n* (%)	73 (28.9)	82 (20.0)	0.206	44 (23.3)	52 (27.5)	0.097
Initial antibiotic use, *n* (%)						
Ampicillin	5 (2.0)	5 (1.2)	0.06	4 (2.1)	2 (1.1)	0.085
Ampicillin/sulbactam	29 (11.5)	52 (12.7)	0.038	25 (13.2)	26 (13.8)	0.015
Piperacillin/tazobactam	48 (19.0)	76 (18.6)	0.01	37 (19.6)	37 (19.6)	< 0.001
First-generation cephalosporin	3 (1.2)	2 (0.5)	0.077	2 (1.1)	2 (1.1)	< 0.001
Third-generation cephalosporin without effect for *Pseudomonas aeruginosa*	38 (15.0)	55 (13.4)	0.045	30 (15.9)	24 (12.7)	0.091
Fourth-generation cephalosporin	6 (2.4)	14 (3.4)	0.063	5 (2.6)	4 (2.1)	0.035
Carbapenem	164 (64.8)	262 (64.1)	0.016	119 (63.0)	116 (61.4)	0.033
Aminoglycoside	5 (2.0)	5 (1.2)	0.06	4 (2.1)	2 (1.1)	0.085
Fluoroquinolone	68 (26.9)	103 (25.2)	0.039	49 (25.9)	54 (28.6)	0.059
Macrolide	43 (17.0)	54 (13.2)	0.106	30 (15.9)	24 (12.7)	0.044
Tetracycline	7 (2.8)	30 (7.3)	0.21	7 (3.7)	5 (2.6)	0.06
Clindamycin	12 (4.7)	20 (4.9)	0.007	8 (4.2)	11 (5.8)	0.073
Anti-MRSA drug	43 (17.0)	44 (10.8)	0.181	23 (12.2)	22 (11.6)	0.016
Antifungal drug	5 (2.0)	5 (1.2)	0.06	4 (2.1)	4 (2.1)	< 0.001

*Abbreviations: rhTM* recombinant human-soluble thrombomodulin, *AT* antithrombin, *SMD* standardized mean difference, *SD* standard deviation, *ICU* intensive care unit, *HCU* high care unit, *MRSA* methicillin-resistant *Staphylococcus aureus*

Table 2 shows the outcomes in the two groups. Before propensity score matching, in-hospital mortality did not differ significantly between the combination therapy group and the monotherapy group (43.5% vs. 47.7%, *p* = 0.298). The proportion of patients requiring red cell transfusion was significantly higher in the combination therapy group compared with the monotherapy group (38.3% vs. 20.0%, *p* < 0.001). After propensity score matching, in-hospital mortality did not differ significantly between the combination therapy group and the monotherapy group (40.2% vs. 45.5%, *p* = 0.350). The proportion of patients requiring red cell transfusion was significantly higher in the combination therapy group compared with the monotherapy group (37.0 vs. 25.9%, *p* < 0.001).

**Table 2:** In-hospital mortality in the unmatched and propensity-matched groups

	Unmatched group			Propensity-matched group		
	rhTM+AT	rhTM	*p*	rhTM+AT	rhTM	*p*
Outcomes	*n* = 253	*n* = 409		*n* = 189	*n* = 189	
In-hospital mortality, *n* (%)	110 (43.5)	195 (47.7)	0.298	76 (40.2)	86 (45.5)	0.350
Proportion of patients with red cell transfusion, *n* (%)	97 (38.3)	82 (20.0)	< 0.001	70 (37.0)	49 (25.9)	< 0.001

*Abbreviations: rhTM* recombinant human-soluble thrombomodulin, *AT* Antithrombin

## Discussion

In this retrospective study using a national inpatient database in Japan, combination therapy with rhTM and AT was not associated with lower in-hospital mortality compared with rhTM monotherapy in patients with severe community-acquired pneumonia-associated DIC. Furthermore, the proportion of patients requiring red cell transfusion was significantly higher in the combination therapy group compared with that in the monotherapy group.

Several possible reasons can be considered for our finding that combination therapy with rhTM and AT was not associated with decreased in-hospital mortality. First, AT may not improve mortality in patients with sepsis-associated DIC, and therefore, the combination therapy may not show a significant difference in outcomes compared with the monotherapy. In a Japanese RCT, AT therapy significantly improved DIC recovery on day 3 compared with the no-treatment group (53.3% vs. 20.0%) but did not improve mortality [35]. A recent systematic review and meta-analysis of 2858 patients with severe sepsis and DIC showed AT was not associated with reduced mortality (risk ratio [RR], 0.95; 95% confidence interval [CI], 0.88–1.03) [22]. Another systematic review of RCTs reached the same conclusion [21].

Therefore, the combination therapy may not be sufficient to improve mortality in patients with sepsis-associated DIC.

Second, combination therapy with rhTM and AT may cause complications such as bleeding complications. A recent systematic review revealed that AT therapy increased bleeding in sepsis patients (RR, 1.58; 95% CI, 1.35–1.84) without reducing mortality (RR, 0.95; 95% CI, 0.88–1.03) [22]. Our study showed that the proportion of patients requiring red cell transfusion was increased in the combination therapy group. Therefore, combination therapy with rhTM and AT may have caused bleeding complications without reducing mortality.

Third, the standard dose of AT used in Japan may not be sufficient to improve mortality. AT needs to reach a plasma concentration activity of 200–250% to show clinically relevant pharmacological activity [36]. In Japan, AT doses of 1500 IU/day or 30–60 IU/kg for a maximum of 3 days are covered by the universal health insurance system for patients with DIC, and AT doses of 1500 IU/day or 30 IU/kg/day are widely used [35]. However, the average plasma AT concentration activity in a previous RCT was 107% after AT administration at 30 IU/kg for 3 days [35]. Similarly, the plasma AT concentration activity may have not increased adequately after AT administration in another report that failed to prove the effectiveness of combination therapy for DIC [17].

Although a recent study suggested that rhTM with or without AT might be associated with better prognosis in the patients with sepsis-induced DIC compared with other DIC treatments [20], the study did not investigate whether rhTM with AT was associated with lower mortality compared with rhTM alone. On the other hand, our results suggest that combination therapy with rhTM and AT may be an ineffective therapeutic approach for reducing mortality and increase bleeding in patients with DIC caused by severe community-acquired pneumonia. Actually, our results are in accordance with those of previous studies, which did not show clinical benefits [17] and showed increase in bleeding complications [12, 21, 22]. Furthermore, additional AT therapy is expensive [37], and thus, avoidance of AT may have a major impact on the treatment costs for DIC.

The strength of the present study was the study design based on a real-world clinical setting. The study included approximately 50% of inpatients who were admitted to acute-care hospitals in Japan.

Our study had several limitations. First, the database lacks clinical records such as severity scores and blood culture results. The specific organism names and serum coagulation data remained unknown. Second, the DPC database also lacks clinical data such as results of laboratory data. Therefore, we could not investigate the serum value of AT% before or after AT administration. Third, we did not investigate the doses of rhTM or AT used. Fourth, the outcome in the present study was blood cell transfusion from 3 days of admission to discharge defined as bleeding events and we excluded the patients who required blood cell transfusion within 2 days of admission. However, the methods may exclude several patients who actually experienced bleeding incidents and required blood cell transfusion within 2 days of admission due to the effects of rhTM and/or AT. Fifth, unmeasured confounders may have biased the results even though we used propensity score matching to adjust for patient background characteristics.

## Conclusions

Compared with rhTM monotherapy, combination therapy with rhTM with AT for severe community-acquired pneumonia-associated disseminated intravascular coagulation may be ineffective for reducing mortality and increase bleeding.

## Supplementary information

**Additional file 1:** **Table S1.** ICD-10 codes to define sepsis **Table S2.** ICD-10 Codes for Identifying Pneumonia and Disseminated Intravascular Coagulation

## Data Availability

Data cannot be made publicly available for ethical reasons as the data are patient data. The data are available to interested researchers upon reasonable request to the corresponding author, pending ethical approval.

## Abbreviations

AT: Antithrombin
CI: Confidence interval
DIC: Disseminated intravascular coagulation
HCU: High care unit
ICD-10: International Classification of Diseases Tenth Revision
ICU: Intensive care unit
JCS: Japan coma scale
MRSA: Methicillin-resistant *Staphylococcus aureus*
RCT: Randomized controlled trial
rhTM: Recombinant human-soluble thrombomodulin
RR: Risk ratio
SD: Standard deviation
SMD: Standardized mean difference

## References

[1] Warren BL, Eid A, Singer P, Pillay SS, Carl P, Novak I (2001). Caring for the critically ill patient. High-dose antithrombin III in severe sepsis: a randomized controlled trial. JAMA.

[2] Bakhtiari K, Meijers JC, de Jonge E, Levi M (2004). Prospective validation of the International Society of Thrombosis and Haemostasis scoring system for disseminated intravascular coagulation. Crit Care Med.

[3] Tagami T, Matsui H, Horiguchi H, Fushimi K, Yasunaga H (2014). Antithrombin and mortality in severe pneumonia patients with sepsis-associated disseminated intravascular coagulation: an observational nationwide study. J Thromb Haemost.

[4] Tagami T, Matsui H, Horiguchi H, Fushimi K, Yasunaga H (2015). Recombinant human soluble thrombomodulin and mortality in severe pneumonia patients with sepsis-associated disseminated intravascular coagulation: an observational nationwide study. J Thromb Haemost.

[5] Nishida O, Ogura H, Egi M, Fujishima S, Hayashi Y, Iba T (2018). The Japanese Clinical Practice Guidelines for Management of Sepsis and Septic Shock 2016 (J-SSCG 2016). J Intensive Care..

[6] Levi M, Toh CH, Thachil J, Watson HG (2009). Guidelines for the diagnosis and management of disseminated intravascular coagulation. Br J Haematol.

[7] Di Nisio M, Baudo F, Cosmi B, D'Angelo A, De Gasperi A, Malato A (2012). Italian Society for Thrombosis and Haemostasis. Diagnosis and treatment of disseminated intravascular coagulation: Guidelines of the Italian Society for Haemostasis and Thrombosis (SISET). Thromb Res.

[8] Rhodes A, Evans LE, Alhazzani W, Levy MM, Antonelli M, Ferrer R (2017). Surviving Sepsis Campaign: International Guidelines for Management of Sepsis and Septic Shock: 2016. Intensive Care Med.

[9] Ito T, Maruyama I (2011). Thrombomodulin: Protectorate God of the vasculature in thrombosis and inflammation. J Thromb Haemost.

[10] Hayakawa M, Yamakawa K, Saito S, Uchino S, Kudo D, Iizuka Y (2016). Recombinant human soluble thrombomodulin and mortality in sepsis-induced disseminated intravascular coagulation. A multicentre retrospective study. Thromb Haemost..

[11] Yamakawa K, Ogura H, Fujimi S, Morikawa M, Ogawa Y, Mohri T (2013). Recombinant human soluble thrombomodulin in sepsis-induced disseminated intravascular coagulation: a multicenter propensity score analysis. Intensive Care Med..

[12] Saito H, Maruyama I, Shimazaki S, Yamamoto Y, Aikawa N, Ohno R (2007). Efficacy and safety of recombinant human soluble thrombomodulin (ART-123) in disseminated intravascular coagulation: results of a phase III, randomized, double-blind clinical trial. J Thromb Haemost.

[13] Yamakawa K, Aihara M, Ogura H, Yuhara H, Hamasaki T, Shimazu T (2015). Recombinant human soluble thrombomodulin in severe sepsis: a systematic review and meta-analysis. J Thromb Haemost.

[14] Yamakawa K, Levy JH, Iba T (2019). Recombinant human soluble thrombomodulin in patients with sepsis-associated coagulopathy (SCARLET): an updated meta-analysis. Crit Care.

[15] Vincent JL, Francois B, Zabolotskikh I, Daga MK, Lascarrou JB, Kirov MY (2019). Effect of a Recombinant Human Soluble Thrombomodulin on Mortality in Patients With Sepsis-Associated Coagulopathy: The SCARLET Randomized Clinical Trial. JAMA.

[16] Sakurai T, Yamada S, Kitada M, Hashimoto S, Harada M, Kimura F (2013). A comparative study of the efficacy of recombinant thrombomodulin monotherapy with that of recombinant thrombomodulin and antithrombin combination therapy for infectious disseminated intravascular coagulation. J Jpn Assoc Acute Med..

[17] Yasuda N, Goto K, Ohchi Y, Abe T, Koga H, Kitano T (2016). The efficacy and safety of antithrombin and recombinant human thrombomodulin combination therapy in patients with severe sepsis and disseminated intravascular coagulation. J Crit Care.

[18] Umemura Y, Yamakawa K, Hayakawa M, Kudo D, Fujimi S (2018). Concomitant Versus Individual Administration of Antithrombin and Thrombomodulin for Sepsis-Induced Disseminated Intravascular Coagulation: A Nationwide Japanese Registry Study. Clin Appl Thromb Hemost..

[19] Iba T, Hagiwara A, Saitoh D, Anan H, Ueki Y, Sato K (2017). Effects of combination therapy using antithrombin and thrombomodulin for sepsis-associated disseminated intravascular coagulation. Ann Intensive Care.

[20] Tanaka K, Takeba J, Matsumoto H, Ohshita M, Annen S, Moriyama N (2019). Anticoagulation Therapy Using rh-Thrombomodulin and/or Antithrombin III Agent is Associated With Reduction in in-Hospital Mortality in Septic Disseminated Intravascular Coagulation: A Nationwide Registry Study. Shock.

[21] Umemura Y, Yamakawa K, Ogura H, Yuhara H, Fujimi S (2016). Efficacy and safety of anticoagulant therapy in three specific populations with sepsis: a meta-analysis of randomized controlled trials. J Thromb Haemost.

[22] Allingstrup M, Wetterslev J, Ravn FB, Møller AM, Afshari A (2016). Antithrombin III for critically ill patients: a systematic review with meta-analysis and trial sequential analysis. Intensive Care Med.

[23] Matsuda S, Fujimori K, Kuwabara K, Ishikawa BK, Fushimi K (2011). Diagnosis Procedure Combination as an infrastructure for the clinical study. Asian Pacific J Dis Manag.

[24] Yamana H, Matsui H, Sasabuchi Y, Fushimi K, Yasunaga H (2015). Categorized diagnoses and procedure records in an administrative database improved mortality prediction. J Clin Epidemiol..

[25] Matsuda S, Fujimori K, Fushimi K (2010). Development of casemix based evaluation system in Japan. Asian Pacific J Dis Manag.

[26] Angus DC, Linde-Zwirble WT, Lidicker J, Clermont G, Carcillo J, Pinsky MR (2001). Epidemiology of severe sepsis in the United States: analysis of incidence, outcome, and associated costs of care. Crit Care Med.

[27] Yamana H, Horiguchi H, Fushimi K, Yasunaga H (2016). Comparison of procedure-based and diagnosis-based identifications of severe sepsis and disseminated intravascular coagulation in administrative data. J Epidemiol.

[28] Mandell LA, Wunderink RG, Anzueto A, Bartlett JG, Campbell GD, Dean NC (2007). Infectious Diseases Society of America/American Thoracic Society Consensus Guidelines on the Management of Community-Acquired Pneumonia in Adults. Clin Infect Dis.

[29] Gando S, Iba T, Eguchi Y, Ohtomo Y, Okamoto K, Koseki K (2006). A multicenter, prospective validation of disseminated intravascular coagulation diagnostic criteria for critically ill patients: comparing current criteria. Crit Care Med.

[30] Chikuda H, Yasunaga H, Takeshita K, Horiguchi H, Kawaguchi H, Ohe K (2014). Mortality and morbidity after high-dose methylprednisolone treatment in patients with acute cervical spinal cord injury: a propensity-matched analysis using a nationwide administrative database. Emerg Med J.

[31] Shigematsu K, Nakano H, Watanabe Y (2013). The eye response test alone is sufficient to predict stroke outcome-reintroduction of Japan Coma Scale: a cohort study. BMJ Open.

[32] Takagi K, Aoki M, Ishii T, Nagashima Y, Narita K, Nakagomi T, et al. Japan Coma Scale as a grading scale of subarachnoid hemorrhage: a way to determine the scale. Neurol Surg. 1998; 26:509–515 [In Japanese]. 9635303

[33] Griswold ME, Localio AR, Mulrow C (2010). Propensity score adjustment with multilevel data: setting your sites on decreasing selection bias. Ann Intern Med.

[34] Austin PC (2009). Balance diagnostics for comparing the distribution of baseline covariates between treatment groups in propensity-score matched samples. Stat Med.

[35] Gando S, Saitoh D, Ishikura H, Ueyama M, Otomo Y, Oda S (2013). Japanese Association for Acute Medicine Disseminated Intravascular Coagulation (JAAM DIC) Study Group for the JAAM DIC Antithrombin Trial (JAAMDICAT). A randomized, controlled, multicenter trial of the effects of antithrombin on disseminated intravascular coagulation in patients with sepsis. Crit Care.

[36] Opal SM (2000). Therapeutic rationale for antithrombin III in sepsis. Crit Care Med..

[37] Ciolek A, Lindsley J, Crow J, Nelson-McMillan K, Procaccini D (2018). Identification of cost-saving opportunities for the use of antithrombin III in adult and pediatric patients. Clin Appl Thromb Hemost.

