# Supplementary material for: The effect of antithrombin added to recombinant human-soluble thrombomodulin for severe community-acquired pneumonia-associated disseminated intravascular coagulation: a retrospective cohort study using a nationwide inpatient database
Source: J Intensive Care. 2020 Jan 13;8:8. doi: 10.1186/s40560-019-0419-8 (PMC6958595; doi:10.1186/s40560-019-0419-8)
Supplement: Supplementary file 1 — Additional file 1: Table S1. ICD-10 codes to define sepsis Table S2. ICD-10 Codes for Identifying Pneumonia and Disseminated Intravascular Coagulation [file 40560_2019_419_MOESM1_ESM.docx]

**Table S1. ICD-10 codes to define sepsis**

| ICD-10 codes |
| --- |
| A00 A02 A03 A04 A05 A08 A09 A15 A16 A17 A18 A19 A20 A21 A22 A23 A24 A25 A27 A28 A30 A31 A32 A33 A34 A35 A36 A37 A38 A39 A40 A41 A42 A43 A44 A46 A48 A50 A49 A51 A52 A53 A54 A65 A66 A67 A69 B35 B36 B37 B38 B39 B40 B41 B42 B43 B44 B45 B46 B47 B48 B49 G00 G01 G02 G03 G04 G05 G06 G07 G08 G09 I30 I33 I80 J01 J02 J03 J04 J05 J06 J13 J14 J15 J16 J17 J18 J44.0 J44.1 J47 J85 J86 K35 K36 K37 K57.02 K57.03 K57.12 K57.13 K57.22 K57.23 K57.32 K57.33 K57.42 K57.43 K57.52 K57.53 K57.82 K57.83 K57.92 K57.93 K61 K65 K63.0 K63.1 K75.0 K75.1 K81.0 N10 N11 N12 N34 N39.0 N41 N70 N71 N72 N73 N74 N75 N76 N77 L03 L04 L08 L88 M00 M86 A49.9 T82.6 T82.7 T83.5 T83.6 T84.5 T84.6 T84.7 T85.7 T81.4 T88.0 |

*Abbreviation:* ICD-10: International Classification of Diseases Tenth Revision.

**Table S2. ICD-10 Codes for Identifying Pneumonia and Disseminated Intravascular Coagulation**

| Pneumonia |
| --- |
| ICD-10 codes: J13 J14 J15 J16 J17 J18 |

*Abbreviation*: ICD-10: International Classification of Diseases Tenth Revision.
